# Orphan rare diseases - The unified airways and its importance for the otorhinolaryngologist

**DOI:** 10.1016/j.bjorl.2022.12.003

**Published:** 2022-12-15

**Authors:** Eulalia Sakano, Jose Dirceu Ribeiro

**Affiliations:** a Department of Ophthalmology - Otorhinolaryngology - Faculty of Medical School, State University of Campinas – (Unicamp), Brazil; b Department of Pediatrics - Faculty of Medical School, State University of Campinas – (Unicamp), Brazil

There is no satisfactory definition of orphan disease among the numerous scientific entities in the world. The scientific community has referred to orphan diseases using two separate but related concepts. The first describes diseases neglected by doctors and the second designates diseases of low incidence and prevalence in the population. On average, patients with rare orphan diseases take more than four years before receiving a correct diagnosis. Before that, they usually receive three or more misdiagnoses and consult with at least five different doctors.^1^

Orphan diseases have drawn increasing attention and there is a need for multi and interdisciplinary care, with the inclusion of the otorhinolaryngologist. Among these rare orphan diseases of genetic origin, Primary Ciliary Dyskinesia (PCD) and Cystic Fibrosis (CF) stand out and are considered of significant importance for the otorhinolaryngologist, due to the high prevalence of upper airway involvement. While CF has been extensively studied, DCP has not.

The concept of United or Unified Airway (UAW) stems from the fact that the upper airways and lower airways, which are anatomically and immunologically related, form a single organ. Both are often comorbid because they reflect manifestations of a single disease related to different parts of the respiratory tract. Thus, as allergic rhinitis is to asthma, which constituted the initial concept of United Airways, chronic rhinosinusitis (CRS) is to CF and PCD. The importance of paranasal sinuses in the contamination of lower airways and the success of the eradication of infections of the two airways, have been recognized in the last two decades.^2^

For the understanding and management of upper airways, the following interventions have been proposed: (i) Nasal irrigation with saline solutions in different concentrations; (ii) topical or oral corticosteroids, (iii) antibiotics (oral, IV and nebulized); (iv) dornase alfa, (v) CFTR (*Cystic Fibrosis Transmembrane Regulator*) protein modulators and (vi) nasosinusal surgery. Although there is a lack of significant evidence for most of these procedures, most of them have been approved for use in united airways management based on the following facts:

• The unified/united airways (UAW) pathway theory believes that the sinuses are a focus of initial bacterial colonization which reaches lower airways in CF and PCD. Lung disease and quality of life in patients with CF and PCD are improved with a more aggressive treatment of the upper airways.
• Almost all patients with CF and PCD have CRS and commitment of the LAW.
• Although chronic rhinosinusitis (CRS) can make the bronchopulmonary manifestations of these diseases worse, the reverse is also true, an interrelation explained by the concept of a unified airway.
• Additionally, nasal polyps have an estimated incidence of 0.1% in children, and their presence may raise suspicion of underlying systemic disease such as cystic fibrosis, primary ciliary dyskinesia.
• The effects of intravenous (IV) antibiotics are substantially smaller on biomarkers of sinonasal disease than on lung inflammatory markers, demonstrating that there are limitations regarding the use of IV antibiotics for CRS in CF and PCD.
• Patients with CF and PCD undergoing surgery for CRS exhibit a substantial decrease in the bacterial community in the paranasal sinuses preventing or delaying aspiration into the lower airways.
• The upper airways are the site of the first colonization and persistence of infection by *Pseudomonas aeruginosa* in CF and PCD, constituting a stimulus for early detection and eradication of microorganisms in united/unified airways.
• About 96% of *Pseudomonas aeruginosa* strains detected in the upper airways and lower airways are genetically identical, both pre and post lung transplantation in patients with CF and PCD, which reinforces the concept of “united airways”.
• Increased chance of eradication of *Pseudomonas aeruginosa* from the sinuses occurs before it changes to a mucoid phenotype and forms biofilms, making eradication nearly impossible.
• Periodic and rigorous monitoring of lower airways and upper airways colonization and an aggressive treatment results in successful and lasting eradication of *Pseudomonas aeruginosa*.
• Pre-lung transplantation sinus surgery may decrease the rate of lower airways infection in patients with CF and PCD.

Among the rare diseases that can cause commitment of the airways, PCD is one of the most significant and its diagnosis is a great challenge for otorhinolaryngologist.

While the diagnosis of CF is broadly defined, the diagnosis of PCD is a problem for all health specialties.^3^, ^4^, ^5^

In recent decades, the investigation of clinical signs and symptoms suggestive of PCD can be performed by: (i) Screening: PICADAR Score and nasal Nitric Oxide (nNO); (ii) Structure: Transmission Electron Microscopy (TEM), Immunofluorescence; (iii) Function: High-Speed ​​Video Microscopy Analysis (HSVMA) with assessment of ciliary beating pattern and frequency and (iv) Genetics: Genome/Exome Sequencing, either whole or a group of genes.

However, all these methods can give false negative results, and the disagreements between the American Thoracic Society (ATS) and the European Respiratory Society (ERS) guidelines regarding the diagnosis of PCD have resulted in confusion for physicians, making confirmation difficult diagnosis. ATS guideline is based on genetic evaluation, nNO and MET, while the ERS guideline focuses on high-speed videomicroscopy analysis (HSVMA), MET, nNO and Immunofluorescence.

Therefore, when considering patients with recurrent or chronic upper and lower airway diseases, the otorhinolaryngologist must establish a differential diagnostic between CF and PCD. The main differences and similarities between these two diseases can be seen in Table 1.

**Table 1:** Main similarities and differences between two rare diseases (Cystic Fibrosis and Primary Ciliary Dyskinesia) which otolaryngologists should consider.

Features	Cystic fibrosis	Primary ciliary dyskinesia
Orphan/rare disease	No/Yes	Yes/Yes
Importance of UAW	Yes	Yes
Genetics: autosomal recessive	Yes	Yes + X-linked
Genes/proteins involved	1	>50
Approximate prevalence	1/2,000 to 1/10,000 NB	1/20,000 NB
Newborn Screening Test (NBST)	Yes	No
Screening	IRT	PICADAR and nNO
Neonatal period	20% Meconium Ileus	80% Respiratory discomfort
Gold standard for diagnosis	Yes	No
Recurring otitis media	No	Yes
Situs inversus/heterotaxy	No	Yes
Multiple organs	Yes ++++	Yes +
Chronic rhinosinusitis (CRS)	Yes - Neutrophilic	Yes – Neutrophilic
Prevalence CRS/Polyps (%)	100/∼30	50/15
Ciliary Dyskinesia	Secondary	Primary
Bronchiectasis	Diffuse	More in the bases
nNO/FeNO	Persistent low	Low in adults
		Normal or low in children
Brazilian guidelines	Yes	No
Compliance with ATS/ERS guidelines	Yes	No
Articles on Pubmed	59,634	5,442

ATS, American Thoracic Society; ERS, European Respiratory Society; nNO, nasal Nitric Oxid; PICADAR, PrImary CiliAry DyskinesiA Rule; CRS, Chronic Rhinosinusitis; UAW, United/Unified Airways; NB, Newborn; FeNo, Fractional Exhaled Nitric Oxide; IRT, Immune Reactive Trypsinogen.

Rare and orphan diseases pose a challenge to all physicians who treat respiratory diseases. The dissemination of knowledge regarding the diagnosis and management of these diseases, among related specialties, can maximize the quality of life of patients.

## Conflicts of interest

The authors declare no conflicts of interest.
